# The “polyglandular crisis” behind recurrent hyponatremia: misdiagnosis of a case of autoimmune polyglandular syndrome type 2 and clinical lessons learned

**DOI:** 10.3389/fimmu.2026.1744295

**Published:** 2026-01-22

**Authors:** Manli Yan, Hai Wu, Jingyun Deng, Yiting Wang, Haoyue Huang, Hua Wei

**Affiliations:** 1 The Second Clinical Medical College, Guangzhou University of Traditional Chinese Medicine, Guangzhou, China; 2 Department of Traditional Chinese Medicine, Meizhou Maternity and Child HealthCare Hospital, Meizhou, China; 3 Department of Endocrinology, Guangdong Provincial Hospital of Chinese Medicine, Guangzhou, China

**Keywords:** Addison’s disease, autoimmune polyglandular syndrome, endocrine function monitoring, Hashimoto’s thyroiditis, hyponatremia, misdiagnosis

## Abstract

Autoimmune polyglandular syndrome (APS) is a group of clinical syndromes resulting from genetic dysfunction of the immune system, affecting multiple endocrine glands as well as non-endocrine organs. Herein, we report a case of APS type 2 (APS-2) in an adult female, characterized predominantly by emaciation, fatigue, palpitations, and notably, refractory hyponatremia. In the late stage of her illness, the patient developed psychiatric abnormalities and was repeatedly hospitalized and treated in neurological facilities; however, no significant abnormalities were detected on relevant examinations. She was subsequently transferred to Guangdong Provincial Hospital of Traditional Chinese Medicine, where a comprehensive endocrine evaluation eventually led to the diagnosis of APS-2. The case was marked by highly non-specific clinical manifestations, which resulted in multiple episodes of misdiagnosis and missed diagnosis throughout her course of treatment. Drawing on the detailed clinical course of this patient and a review of relevant literature, this article analyzes the clinical heterogeneity, diagnostic challenges, and reasons for misdiagnosis associated with APS-2. Furthermore, it highlights the importance of dynamic monitoring of multiple glandular functions, enhancing clinicians’ recognition of this syndrome, and multidisciplinary collaboration to improve patient outcomes and reduce delays caused by misdiagnosis.

## Introduction

Autoimmune polyendocrine syndrome (APS) (1) is a group of clinical syndromes caused by genetic dysfunction of the immune system, which can manifest as simultaneous or sequential dysfunction of multiple endocrine or non-endocrine glands (2).

Based on pathogenesis and clinical characteristics, APS can be classified into type 1 (APS-1), type 2 (APS-2), and IPEX syndrome (3). APS-1 is more common in children and is mainly caused by mutations in the AIRE gene (4), which lead to immune tolerance defects and abnormal clonal proliferation of immune cells. The classic triad of APS-1 includes chronic mucocutaneous candidiasis, primary adrenocortical insufficiency, and hypoparathyroidism (4, 5). APS-2 occurs more frequently in adults (6) and is usually associated with certain HLA haplotypes, such as HLA-DR3 and HLA-DR4 (6). Its typical manifestations (7) include autoimmune adrenal insufficiency, autoimmune thyroid disease, and type 1 diabetes mellitus, and it may also be accompanied by various other autoimmune disorders.

The clinical manifestations of APS are highly complex and heterogeneous (8), with significant individual differences in the types of glands affected, the extent of glandular dysfunction, and the specific symptoms presented. Early-stage APS often lacks distinctive features, making misdiagnosis or missed diagnosis common and potentially leading to delays in treatment and increased healthcare costs. Here, we report a case of APS-2 admitted to Guangdong Provincial Hospital of Traditional Chinese Medicine in March 2022, in which emaciation, fatigue, and palpitations were the initial presentations, with refractory hyponatremia as a prominent feature. By reviewing the patient’s repeated misdiagnoses and treatment course and analyzing relevant literature, this article aims to enhance clinical awareness of the syndrome’s diversity and the importance of early recognition, thereby promoting standardized diagnosis and management.

## Case presentation

A female patient in her early 50s was admitted to the Department of Endocrinology at Guangdong Provincial Hospital of Chinese Medicine in March 2022 with a history of weight loss for more than one year, recurrent fatigue and palpitations for over four months, which had worsened over the past five days.

The patient experienced progressive, unexplained weight loss beginning one year prior to admission, without other significant discomfort at that time, and did not seek further medical attention. At the end of 2021, she developed fatigue, episodes of palpitations, occasional chest tightness, and vomiting. The vomitus was moderate in amount, non-projectile, and not coffee-ground in appearance. She did not report dizziness or headache. She subsequently presented to the First Affiliated Hospital of Guangzhou University of Chinese Medicine, where laboratory tests revealed a serum sodium (Na^+^) of 110 mmol/L and chloride (Cl^−^) of 83.5 mmol/L. She was diagnosed with electrolyte disturbance, and symptoms slightly improved with symptomatic treatment; she was discharged at the request of the patient and her family.

On December 26, 2021, she continued to experience palpitations, nausea, vomiting, and developed psychiatric and consciousness disturbances, including episodes of delirium. To rule out psychiatric causes, she was admitted to the Department of Neurology at the Brain Hospital of Guangzhou Medical University. Relevant tests showed Na^+^ of 120 mmol/L, Cl^−^ of 90 mmol/L, with no abnormalities found in special electroencephalography, sex hormone assessment, immunological panel, or vasculitis screening. The diagnoses were hyponatremia and metabolic encephalopathy (Hyponatremia can disrupt the osmotic balance in the central nervous system, potentially serving as a primary contributor to the development of neurological and psychiatric abnormalities in affected patients.). After supportive treatment, her mental status improved, and upon discharge, Na^+^ was 132 mmol/L.

Approximately two weeks prior to this admission, she again experienced fatigue, headache, vomiting, and palpitations, and was re-admitted to the same neurology department. Repeat laboratory evaluations indicated Na^+^ 110 mmol/L, Cl^−^ 83.5 mmol/L, morning serum cortisol (08:00) of 68.7 nmol/L (reference range 145.4–619.4 nmol/L), and ACTH (08:00) of 190.4 pmol/L (reference range 1.60–13.90 pmol/L). EEG and lumbar puncture were unremarkable. She was diagnosed with tension-type headache, autonomic dysfunction, and hyponatremia/hypochloremia. She received symptomatic treatment for insomnia and anxiety. Her symptoms improved after treatment, and she was discharged with a recommendation for outpatient endocrine follow-up.

Five days prior to the current admission, her previous symptoms recurred and she presented to our outpatient clinic. She was admitted with a provisional diagnosis of “electrolyte disturbance (hyponatremia, hypochloremia) for further evaluation.” Over the past year, her weight had decreased by approximately 8 kg.

Her past medical history was notable for hyperthyroidism combined with Hashimoto’s thyroiditis for over six years, which was treated regularly with propylthiouracil (usually 50–100 mg) and occasionally with levothyroxine. She discontinued all medications at the end of 2019 under physician guidance and had not undergone regular follow-up since. She denied a history of diabetes or other chronic medical illnesses.

On admission, the patient was alert but complained of fatigue, generalized weakness, palpitations, chest tightness, and dizziness. She denied headache, abdominal distension, or abdominal pain. She also reported poor sleep quality, polyuria, and normal bowel movements. Her BMI was 17.5 kg/m², and she appeared emaciated. Hyperpigmentation of the skin and mucous membranes was observed, most prominently on the face, tongue, oral mucosa, palmar creases, and around the joints. Her skin exhibited reduced elasticity and moisture. No edema was noted in either lower limb.

**Figure 1:**
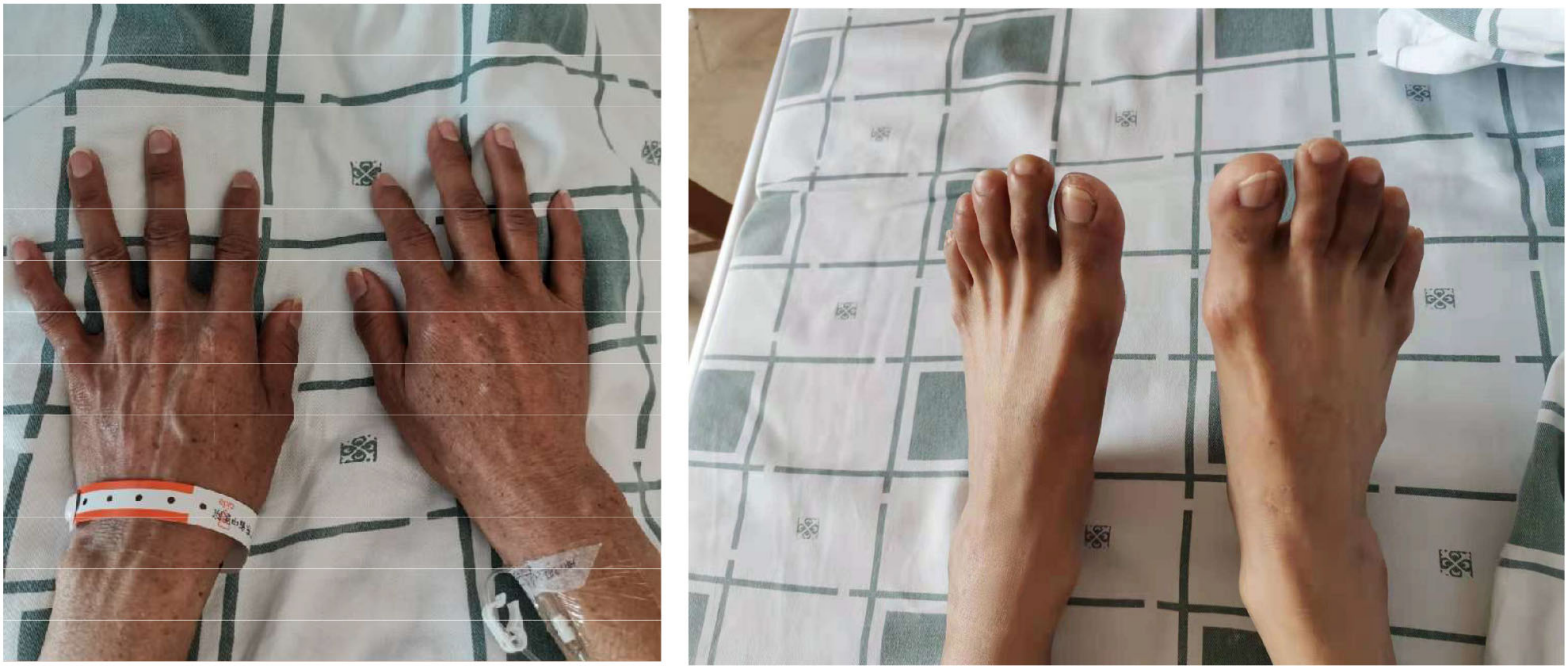
Hyperpigmentation of the patient’s hands and feet.

Given that the patient was characterized by refractory hyponatremia and had multiple hospitalizations at outside hospitals, where initial screening revealed abnormal serum cortisol and adrenocorticotropic hormone levels, and physical examination showed hyperpigmentation of the skin (**Figure 1**), along with a history of thyroid dysfunction, a comprehensive evaluation of all endocrine axes was recommended upon this admission. Detailed test results are shown in the accompanying **Table 1**. Blood glucose levels and electrocardiogram findings were unremarkable, and parathyroid hormone levels were within the normal range.

**Table 1:** Laboratory test results of the patient.

Parameters	Patient	Reference range
Serum electrolytes test		
Na^+^, mmol/L	116	137-147
Cl^-^, mmol/L	86.1	99-110
K^+^, mmol/L	4.24	3.5-5.3
Mg^2+^, mmol/L	0.7	0.75-1.02
P, mmol/L	1.60	0.85-1.51
Gonadal hormone		
Testosterone, nmol/L	<0.09	0.10-1.42
Estradiol, pmol/L	<18.35	18.4-5050
Progesterone, nmol/L	1.94	0.0-0.4
Prolactin, mIU/L	646.1	97.7-651.7
Follicle stimulating hormone, IU/L	105.4	25.8-134.8
Luteinizing hormone, IU/L	63.08	7.7-58.5
Thyroid hormones and associated antibodies		
Free triiodothyronine, pmol/L	4.97	3.50-6.50
Free thyroxine, pmol/L	9.15	11.50-22.70
Thyroid- stimulating hormone, mIU/L	9.153	0.51-4.97
Thyroglobulin antibodies, U/ml	>500	<60
Thyroid peroxidase antibody, U/ml	>1300	<60
Thyrotropin receptor antibodies, IU/L	3.14	<1.75
COR		
COR(7:00-9:00),nmol/L	51.1	145.4-619.4
COR(15:00-17:00),nmol/L	54.1	94.9-462.4
COR(0:00),nmol/L	45	
ACTH		
ACTH(7:00-9:00),pmol/L	329.61	1.60-13.90
ACTH(15:00-17:00),pmol/L	208.37	1.60-13.90
ACTH(0:00),pmol/L	159.30	1.60-13.90
Three hypertension-related parameters(measured in supine position)		
Angiotensin II, pg/ml	71.31	25.00-129.00
Aldosterone, pg/ml	75.00	10.00-160.00
Renin, pg/ml	45.05	4.00-24.00
Aldosterone/Renin	1.66	0.00-25.00
Urinary electrolyte concentrations		
Urinary potassium concentration, mmol/L	10.51	16.70-66.70
Urinary phosphate concentration, mmol/L	4.50	12.90-43.90
Urinary chloride concentration, mmol/L	52.8	113.3-170.0
Urinary sodium concentration, mmol/L	62	86.7-173.7
Urinary calcium concentration, mmol/L	1.30	1.70-5.30
24-hour urinary electrolyte excretion		
24-hour urinary potassium excretion, mmol/24h	36.00	25-125
24-hour urinary phosphate excretion, mmol/24h	15.41	13.0-24.0
24-hour urinary chloride excretion, mmol/24h	181	170-250
24-hour urinary sodium excretion, mmol/24h	212	40-220
24-hour urinary calcium excretion, mmol/24h	4.45	1.7-5.3
24-hour urine volume, ml/24h	3425	1000-2000
Urine osmolality, mOsm/kg H_2_O	545	600-1000

COR, cortisol; ACTH, adrenocorticotropic hormone.

**Figure 2:**
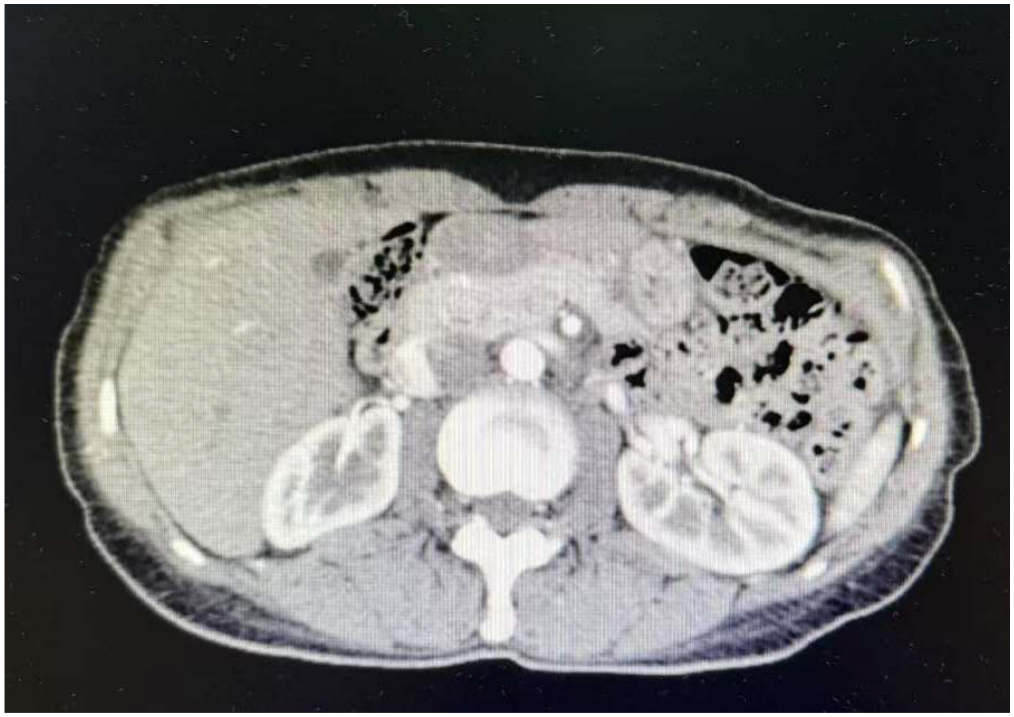
Contrast-enhanced CT of the adrenal glands. No obvious abnormalities were observed in the kidneys or adrenal glands. Multiple high-density lesions were detected in S5 of the liver, which were considered to be intrahepatic bile duct stones or calcifications.

**Figure 3:**
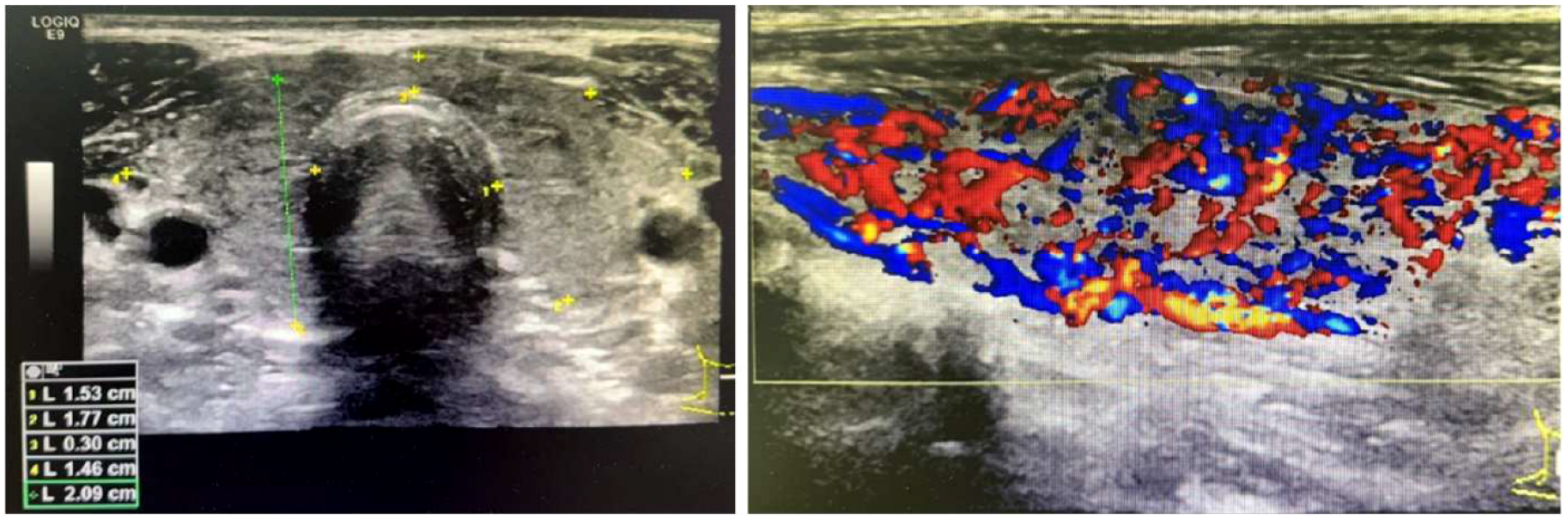
Thyroid ultrasonography. The thyroid gland was mildly enlarged with diffuse parenchymal echotexture abnormalities.

**Figure 4:**
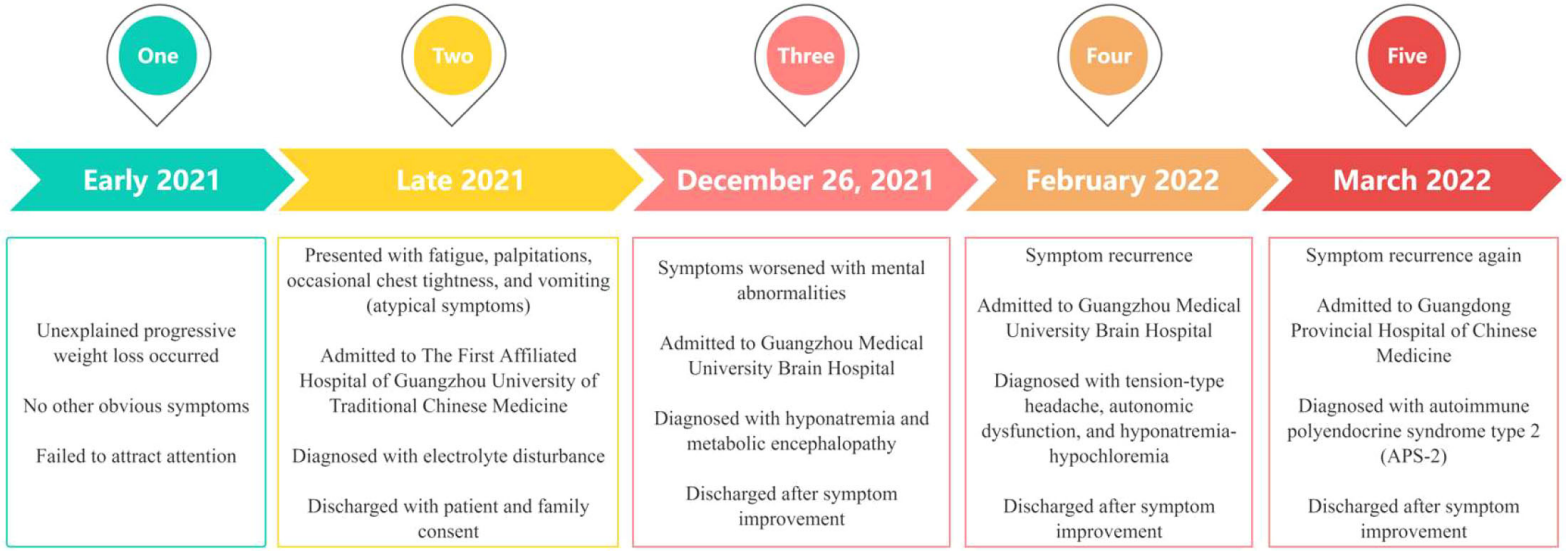
Timeline of the patient’s clinical course, key laboratory results, and diagnoses.

Based on the above laboratory and imaging findings (**Table 1**; **Figures 1**–**4**), the diagnoses of Addison’s disease(Elevated ACTH levels, reduced cortisol, along with typical skin and mucosal hyperpigmentation, in the presence of a normal adrenal morphology that excludes organic pathology, support the diagnosis of autoimmune-mediated adrenal damage.), Hashimoto’s thyroiditis(Positive thyroid-related antibodies.), hypothyroidism(Decreased FT4 and elevated TSH levels.), and intrahepatic bile duct stones were made. On March 14, 2022, hydrocortisone replacement therapy was initiated at a dose of 20 mg once daily in the morning, along with symptomatic treatment. Considering the patient was markedly emaciated (BMI: 17.5 kg/m²) with a body weight of only 39.5 kg, the required hydrocortisone dose calculated by body weight was just 13.825 mg (0.35 mg/kg); therefore, on March 16, the dose was adjusted to hydrocortisone 10 mg once daily in the morning. After initiating hydrocortisone therapy, multiple reexaminations revealed that the serum sodium level gradually stabilized at around 130 mmol/L, and symptoms of fatigue, weakness, and palpitations were alleviated, indicating the effectiveness of the treatment regimen. The patient was discharged with medication and instructed to take the medication regularly and attend follow-up visits as scheduled.

At the outpatient follow-up on April 25, 2022, the patient continued hormone replacement therapy. Re-examination showed a morning serum cortisol level (7–9 am) of 253.6 nmol/L; serum electrolytes were as follows: Na^+^ 136 mmol/L, Cl^−^ 104 mmol/L. The patient reported no significant discomfort and had not experienced any further episodes of altered mental status. Improvement in symptoms correlates with the normalization of laboratory parameters, further confirming the effectiveness of the diagnosis and treatment plan.

## Discussion

Most previously reported cases of APS-2 have demonstrated a chronic and progressive course (3). However, the clinical manifestations of APS-2 are highly heterogeneous, with significant individual variation in the type and extent of glandular involvement. Symptoms are often nonspecific, presenting a considerable challenge for clinical diagnosis and treatment. Hormones, electrolytes, and nutrients are not only fundamental to maintaining normal physiological function, but also play a critical role in determining physical well-being and emotional status. Therefore, when endocrine gland dysfunction occurs, patients may present with both classic “endocrine symptoms” and atypical manifestations (9). For example, Glick, L. R. et al. (10) reported a case of APS-2 that initially presented with cardiac tamponade. Zihong Yao et al. (6) described a case of APS-2 complicated by adrenal crisis, in which persistent fatigue was the main clinical feature. Due to the lack of specificity, the diagnosis was delayed by nine years; In the present case, the patient’s main clinical symptoms were emaciation, fatigue, and palpitations, along with refractory hyponatremia. The late stage of the disease was further complicated by psychiatric abnormalities—none of which are typical features—thus greatly increasing the difficulty of diagnosis.

Based on this patient’s clinical course and examination results, the main reasons for misdiagnosis are as follows: First, the etiology of hyponatremia was not considered comprehensively. During the initial visit, the patient’s history of vomiting led clinicians to attribute the hyponatremia solely to gastrointestinal loss, overlooking the fact that weight loss and fatigue had already occurred prior to the onset of vomiting. Second, the patient’s symptoms recurred after symptomatic treatment, and even progressed to psychiatric disturbances in the later stage. Clinically, there was a lack of timely recognition of the refractory nature of the hyponatremia and its potential dangers, and hyponatremia was not used as the central clue for further etiological investigation. Instead, diagnostic efforts became focused on exploring central nervous system etiologies of psychiatric abnormalities, neglecting the underlying causes of recurrent hyponatremia, which led to further diagnostic delay. Furthermore, there was insufficient comprehensive analysis of the abnormal signs and test results. During previous hospitalizations, abnormalities in blood cortisol and ACTH levels were observed, and physical examination revealed significant emaciation and skin hyperpigmentation. However, these key clues were overlooked, and the absence of multidisciplinary consultation further delayed the diagnosis.

When hyponatremia cannot be explained by common causes, the differential diagnosis should focus on blood volume status and urinary markers. Syndrome of inappropriate antidiuretic hormone secretion is characterized by normovolemic hyponatremia, with elevated urinary sodium and osmolality. Before making a diagnosis, adrenal and thyroid dysfunction must be excluded, with hypothyroidism confirmed by elevated TSH levels. Hyponatremia caused by gastrointestinal disorders (especially with significant fluid loss due to vomiting or diarrhea) is typically hypovolemic, often accompanied by decreased urinary sodium and hypokalemia. Psychogenic polydipsia results in dilutional hyponatremia, with extremely low urinary osmolality as a hallmark. In the differential process, it is crucial to focus on the core features of each disease, prioritizing the exclusion of life-threatening Addison’s disease. Cortisol and ACTH tests should be performed promptly, and if autoimmune hypothyroidism or diabetes is also present, there should be a high suspicion of APS-2.

It is important to emphasize that endocrine gland function can undergo dynamic changes as the disease progresses or the immune status alters, with some patients experiencing multistage, multidirectional functional abnormalities. In this case, the patient had a history of Hashimoto’s hyperthyroidism, and the current hospitalization revealed hypothyroidism upon reexamination. Despite significant weight loss, the glandular function was not re-evaluated in a timely manner, which highlights this characteristic. Immunological factors have been identified as important mechanisms influencing both endocrine gland function (11, 12) and endocrine metabolism (13), and may result in injury to multiple endocrine glands during acute or multiple phases of illness (14, 15). These findings further highlight the importance of dynamic follow-up and regular monitoring of endocrine function.

Even in patients with chronically progressive APS-2, it is essential to regularly evaluate the function of each endocrine gland. Because multiple glands may be affected simultaneously, interactions can occur between different hormone replacement or regulatory therapies; neglecting underlying glandular abnormalities may lead to serious complications. Tsinopoulou, V. R. et al. (16) reported the case of a 13-year-old girl with a history of Hashimoto’s thyroiditis and hypothyroidism who had been on long-term levothyroxine therapy without regular monitoring of related hormone levels. As her disease progressed, she developed marked emaciation and oligomenorrhea, and preliminary investigations revealed a shift from hypothyroidism to hyperthyroidism. At the time of presentation, she also showed signs of tachycardia and dehydration. Further testing indicated serum electrolyte imbalance, extremely low cortisol levels, and markedly elevated ACTH. Fortunately, timely adjustment of her treatment regimen prevented the occurrence of adrenal crisis. After more comprehensive evaluation, she was ultimately diagnosed with APS-2. In addition, Balachandran, D. M. et al. (17) reported a case of APS-2 in which the patient developed adrenal crisis during late pregnancy, highlighting the need for enhanced dynamic assessment and management of glandular function during special physiological stages.

It should be noted that, in addition to autoimmune-related endocrine diseases, attention must also be paid to newly emerging autoimmune reactions and drug-induced endocrine dysfunctions. In recent years, with the widespread use of immune checkpoint inhibitors (ICPI) and similar therapies, the associated endocrine toxicities have become increasingly recognized. Thomsen, M. J. et al. (14) reported a case of thyroid dysfunction following ICPI therapy, which initially presented as thyrotoxicosis and subsequently progressed to hypothyroidism. Qin Pan et al. (18) reported a case in which the full triad of APS-2 (autoimmune thyroid disease, type 1 diabetes mellitus, and Addison’s disease) was induced after ICPI therapy. At 25 weeks post-treatment, the patient developed hypothyroidism with elevated related antibodies, and at 45 weeks, she experienced primary adrenal insufficiency with adrenal crisis, as well as fulminant type 1 diabetes mellitus accompanied by ketoacidosis. These cases highlight that APS-2 can progress rapidly within a short period and seriously endanger patients’ lives, warranting heightened clinical vigilance.

Given the established association between APS-2 and specific alleles such as HLA-DR3/DR4 (6), HLA genotyping was recommended during the clinical diagnostic process to better clarify the patient’s genetic background and enhance diagnostic accuracy. However, the patient and their family, concerned about the cost of the test and believing that the results would not directly impact the clinical treatment plan, ultimately declined to undergo the testing. As a result, the final diagnosis in this case was based solely on the patient’s clinical symptoms, physical examination, and laboratory test results, without molecular genetic verification of the disease’s genetic susceptibility. This represents a limitation of the study.

After identifying the underlying cause, the management of glandular function (including the choice of hormones, as well as dosage and frequency) should be comprehensively assessed based on the individual patient’s condition, aiming to align with the physiological circadian rhythm of cortisol secretion. The treatment plan for this patient was somewhat unique: considering the patient’s emaciated physique, and on the one hand, the initial symptoms primarily consisted of hyponatremia and fatigue, without prominent signs of aldosterone deficiency such as hyperkalemia or orthostatic hypotension, suggesting a relatively mild requirement for mineralocorticoids. On the other hand, a single morning dose regimen not only mimicked the physiological peak of cortisol secretion in the early morning but also significantly improved the patient’s long-term medication adherence, reducing the risk of missed doses due to frequent dosing. Ultimately, a single morning dose of 10 mg hydrocortisone was chosen, without the addition of fludrocortisone for synergistic therapy.

## Conclusion

APS-2 is characterized by marked clinical heterogeneity and complexity, with significant variation in the types and extent of glandular involvement. Some cases may progress rapidly within a short period, posing a severe threat to patient survival. Therefore, in clinical practice, it is essential to maintain a high index of suspicion for atypical presentations of APS-2 and to prioritize early recognition and systematic evaluation in order to minimize the risks of misdiagnosis and diagnostic delays. In addition, dynamic and standardized follow-up and monitoring of endocrine gland function are critically important. Only through regular assessment of hormone levels and the timely detection of functional changes can individualized and evidence-based treatment plans be developed for patients, reducing the incidence of serious complications and truly safeguarding patient safety and quality of life. This study has certain limitations: genetic testing, such as HLA genotyping, was not conducted, preventing molecular-level verification of genetic susceptibility to APS-2. Future studies could incorporate this data to further elucidate the disease mechanisms.

## Data Availability

The original contributions presented in the study are included in the article/supplementary material. Further inquiries can be directed to the corresponding authors.
